# Prevalence and Risk Factors of Transfusion‐Transmitted Infections Among Blood Donors in a Tertiary Hospital in Kenya

**DOI:** 10.1155/ghe3/6646626

**Published:** 2026-05-18

**Authors:** Collince Odiwuor Ogolla, Bernard Guyah, Apollo O. Maima, Benson Amayo

**Affiliations:** ^1^ School of Public Health & Community Development, Maseno University, P.O. Box 3275-40100, Maseno, Kenya, maseno.ac.ke; ^2^ School of Pharmacy, Maseno University, P.O. Box 3275-40100, Maseno, Kenya, maseno.ac.ke; ^3^ Equity Afya Hospital, P.O. Box 75104-00200, Nairobi, Kenya

**Keywords:** donor, prevalence, transfusion-related infections

## Abstract

**Background:**

Most health‐related conditions depend on blood transfusions for their care. However, transfusion‐related infections (transfusion‐transmitted infections [TTIs]) remain one of the most important concerns in the area of health care across settings of resource‐poor regions.

**Objective:**

The study aimed to determine the status of TTI and some of the risks related to donated blood.

**Methods:**

The study involved 347 participants who donated blood voluntarily to participate in a cross‐sectional research study. Blood samples were tested for hepatitis B, hepatitis C, HIV, syphilis, and malaria. Structured questionnaire was used to gather demographic information and health history data. Data analysis was done through descriptive statistics while using Fisher’s exact test to evaluate exploratory associations.

**Results:**

The prevalence of TTIs was low with hepatitis B occurring in 1.85% of blood donations and malaria in 2.78% of blood donations. The testing process identified no instances of HIV, syphilis, or hepatitis C. The study could only analyze associations between variables because there were so few positive cases.

**Conclusion:**

The prevalence of TTIs among blood donors was low. More extensive multicenter research is necessary to determine risk factors and enhance blood safety protocols.

## 1. Introduction

Blood transfusion is a key component of contemporary healthcare systems. The procedure serves as an essential treatment method for patients suffering from severe anemia along with trauma and surgical blood loss and various hematological disorders [1, 2]. The medical procedure of transfusion saves lives, but it also exposes patients to potential risks, including the transmission of infectious agents such as hepatitis B virus (HBV), hepatitis C virus (HCV), human immunodeficiency virus (HIV), syphilis, and malaria. [3, 4]. Transfusion‐transmitted infections (TTIs) are a significant public health problem, particularly in low‐ and middle‐income countries, due to inadequate screening systems and weak healthcare infrastructure. [5, 6]. The WHO estimates that on an average, millions are exposed to blood‐borne pathogens by transfusion all over the world, particularly in areas where there is a high incidence of infectious diseases and limited availability of safe and voluntary blood donors [7, 8]. The sub‐Saharan African region, including Kenya, faces a substantial burden of transfusion‐transmitted infections (TTIs) due to inadequate donor screening processes, limited availability of reliable testing methods, and a high prevalence of malaria and viral hepatitis. [9, 10]. The hepatitis B vaccine became part of Kenya’s national vaccination program which operates through the Expanded Programme on Immunization (EPI) in 2002 to provide vaccines for all infants [11, 12]. The vaccine has led to decreasing HBV rates among younger people which has helped scientists to understand how different age groups present infection rates.

The WHO blood safety framework served as the main direction for this research, which requires blood to undergo complete testing for all transfusion‐transmissible infections according to the specific local disease patterns [13, 14]. *Plasmodium spp.* transfusion‐transmitted malaria caused by *Plasmodium* species represents an underrecognized but significant safety concern in western Kenya, where malaria is endemic. Consequently, the researchers selected malaria as a primary disease of interest, alongside HBV, HCV, HIV, and syphilis [15]. The Kenyan government has established national blood transfusion regulations which require blood testing to occur at central facilities while educating donors about safe practices. The research discovered different infection patterns showed by donor profiles across various regions, which required researchers to conduct specific studies that would reveal particular dangers and developments within their area of study [16, 17].

The research conducted in other Kenyan locations revealed TTI positivity rates that ranged from 2% to more than 10% based on the demographic characteristics tested, the testing methods employed, and the regional disease burden [18]. The absence of registered data for blood donors creates a local understanding gap which hinders effective intervention efforts. The studies on TTI occurrences have produced multiple research works, but only a small number of studies have investigated the demographic and behavioral risk elements which increase blood donor infection risks in this specific research setting. This study aimed to measure the rate of transfusion‐related infections among blood donors while identifying the demographic and behavioral risk elements which led to these infections. The identification of these elements provides essential information which helps to enhance blood safety measures used in transfusion services and develop donor selection guidelines and decrease infection transmission through blood donations.

## 2. Methods

### 2.1. Study Design

The study was cross‐sectional in design. Participants were recruited using a consecutive sampling approach, whereby all eligible donors presenting during the study period were invited to participate. The study was designed to provide preliminary prevalence estimates rather than to establish causal relationships between risk factors and infections. This study was reported in accordance with the STROBE guidelines.

### 2.2. Study Population

The hospital blood donors who fulfilled all requirements established for study participation were recruited for the research. The study included 347 blood donors as participants. The sample size was determined pragmatically based on the average annual donor pool with the aim of providing prevalence estimates with acceptable precision for low‐frequency outcomes such as TTIs. Participants had to be willing to donate blood, as well as meet the standard eligibility requirements of the hospital for blood donation in order to be included. Donors who had transfusion‐related infections or received blood transfusions in the previous 12 months were excluded from the study, while those who did not want to participate were also left out.

### 2.3. Inclusion Criteria and Exclusion Criteria

Donors who voluntarily donated blood and gave informed consent were included in the study. The study excluded participants who had already disclosed their TTI history or who had received a blood transfusion during the last 12 months or who had chosen not to participate in the research study.

## 3. Data Collection

Demographic factors (age and gender) and behavioral risk factors (i.e., a history of recent surgery, multiple blood donations, a history of unscreened health assessments, etc.) were documented from each donor through a structured questionnaire. These were potential risk factors identified many years ago as related to transfusion‐related infections. The data were collected by trained research assistants in order to achieve the desired consistency and accuracy. Blood samples for all participants were screened against five common transfusion‐related infections: hepatitis B, hepatitis C, HIV, syphilis, and malaria. All tests were done using standard diagnostic methods and laboratory conditions. Specifically, hepatitis B and hepatitis C were screened using enzyme immunoassays (EIAs), HIV was tested using rapid diagnostic tests (RDTs), syphilis was detected using the rapid plasma reagin (RPR) test, and malaria was diagnosed through microscopic examination of blood smears or RDTs. The variable “donor without prior medical screening” was self‐reported during the predonation interview, before laboratory testing, to avoid any possibility of reverse causation. The primary outcome of interest was detection of any transfusion‐related infection in the blood samples.

Donor safety was monitored during and after the donation process. A few mild adverse events, such as fatigue and dizziness, were reported but were transient and did not require medical intervention.

### 3.1. Statistical Analysis

Data analysis was conducted with R Statistical Software Version 3.4.3. Descriptive statistics were used to present demographic data and to show how common TTIs occurred. The study could not perform inferential statistical analysis because there were only five infection cases to investigate. Fisher’s exact test was used to examine the relationships between categorical variables because it works well with small groups and limited information. Regression or correlation analyses were not conducted as there were not enough outcome events to support those tests.

### 3.2. Ethical Consideration

The study was conducted in accordance and adherence with the ethical principles of the Declaration of Helsinki. Confidentiality and anonymity were strictly maintained throughout the study.

## 4. Results

### 4.1. Demographic Characteristics of Study Participants

A total of 347 blood donors were included in the study. The distribution of participants by sex and age group, along with infection status, is presented in Table 1.

**TABLE 1:** Demographic characteristics and infection status of blood donors (*n* = 347).

Variable	Category	Total *n* (%)	Infected *n* (%)	Uninfected *n* (%)
Sex	Male	222 (64.0)	3 (1.35)	219 (98.65)
	Female	125 (36.0)	2 (1.60)	123 (98.40)

Age group (years)	18–30	174 (50.1)	2 (1.15)	172 (98.85)
	31–45	129 (37.2)	3 (2.33)	126 (97.67)
	≥ 46	44 (12.7)	0 (0.00)	44 (100.00)

Overall, 5 donors (1.44%) were found to have at least one TTI.

### 4.2. Prevalence of TTIs

The overall prevalence and distribution of TTIs are presented in Table 2.

**TABLE 2:** Prevalence of transfusion‐transmitted infections among blood donors (*n* = 347).

Infection type	Number of positive cases (*n*)	Prevalence (%)
Hepatitis B	2	1.85
Hepatitis C	0	0.00
HIV	0	0.00
Syphilis	0	0.00
Malaria	3	2.78

**FIGURE 1:**
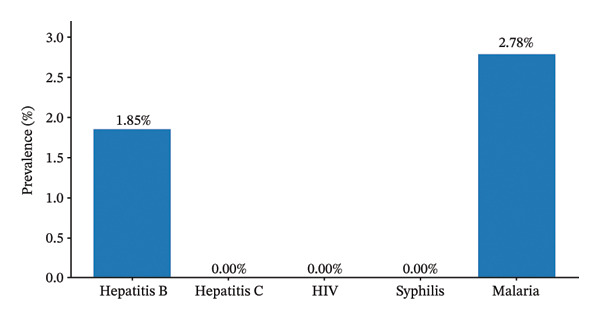
Prevalence of transfusion‐related infections among blood donors. Bar chart showing the prevalence of transfusion‐related infections among blood donors. Malaria had the highest prevalence (2.78%), followed by hepatitis B (1.85%). No cases of HIV, hepatitis C, or syphilis were detected.

Malaria (2.78%) and hepatitis B (1.85%) were the only infections detected. No cases of HIV, hepatitis C, or syphilis were identified (Figure 1).

### 4.3. Exploratory Analysis of Infection by Demographic Factors

**FIGURE 2:**
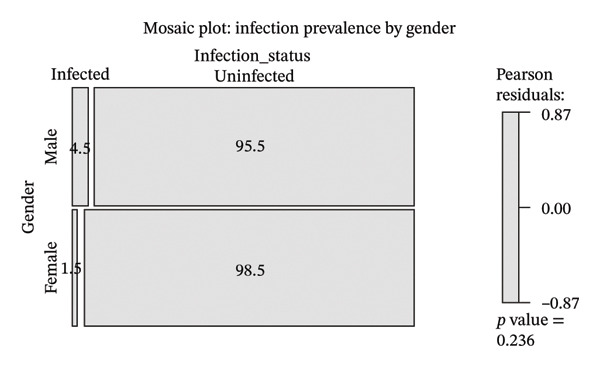
Fisher’s exact test for gender and infection prevalence. Fisher’s exact test indicates a significant association between male gender and infection prevalence (*p* = 0.03), confirming higher infection risk among males, particularly when sample sizes are small.

Given the small number of infection cases (*n* = 5), formal inferential statistical modeling was not appropriate. However, exploratory comparisons were conducted using Fisher’s exact test (Table 3 and Figure 2).

**TABLE 3:** Exploratory association between demographic factors and infection status using Fisher’s exact test.

Variable	Category	Infected n	Uninfected n	*p* value
Sex	Male	3	219	0.03
	Female	2	123	

Age group (years)	18–30	2	172	0.21
	31–45	3	126	
	≥ 46	0	44	

Although a higher proportion of infections was observed among male donors, this finding should be interpreted cautiously due to the very small number of infection cases.

## 5. Discussion

The research establishes fundamental information regarding how common TTIs occur among blood donors. The overall prevalence of TTIs was low (1.44%), with only hepatitis B (1.85%) and malaria (2.78%) detected. The study found no cases of HIV or hepatitis C or syphilis. The facility demonstrates a positive blood safety record according to the findings, but the results need careful assessment because only a small number of infections were discovered.

Blood donors in Kenya and throughout sub‐Saharan Africa exhibit TTI prevalence rates that range from 2% to 10% according to multiple studies. The present study demonstrates lower TTI rates because of successful donor selection methods and better screening methods and greater public understanding of transfusion safety. The results show that the small sample size caused the study to underestimate actual rates of occurrence.

Hepatitis B was found in 1.85% of donors, which is lower than estimates from other Kenyan studies that found blood donor prevalence at approximately 2% or higher [19]. The hepatitis B vaccination program that started in 2002 with the EPI has produced low rates of hepatitis B disease [20, 21]. Vaccination coverage has also decreased the number of people who can be infected by the disease especially in younger age groups. The present study did not assess vaccination status directly; therefore, future studies must investigate age‐specific vaccination data.

The study found malaria as the most common infection which occurred in 2.78% of cases because the disease still poses a transfusion risk in areas where it exists. Blood donors in malaria‐endemic regions of sub‐Saharan Africa show asymptomatic parasitemia according to previous studies [22, 23] which demonstrate similar results. Blood screening protocols do not include malaria as a standard test in Kenya. The study detected malaria to demonstrate the requirement for specific local methods which combat transfusion‐related malaria transmission in areas with high transmission rates especially in Western Kenya.

The exploratory analysis found that male donors showed higher rates of infection than female donors while the 31 to 45 age group exhibited slightly elevated infection rates. Male donors usually make up a larger portion of the donor base in other studies, which also showed that their work activities, daily activities, and social contacts results in different levels of contact with HIV [24–26]. The present study shows infection cases at such low levels that researchers need to exercise strong caution about their results. The study did not have enough power to show significant demographic differences while researchers could not make any causal or inferential deductions from the data.

The study focused on identifying TTIs without establishing their independent risk factors. Regression analysis for inferential statistics was not applied because the study had only five outcome events. The patterns that researchers found in their studies should be regarded as exploratory research because they need further investigation through hypothesis testing. The identification of risk factors and the determination of causal relationships need to be assessed through research that includes multiple study sites.

The study results show that people in this research group do not have HIV, hepatitis C, or syphilis which demonstrates that current screening methods work effectively. The study results show that these infections exist in the general population, but they do not show up in this specific sample of research participants. The organization requires ongoing monitoring together with complete compliance to their established screening procedures. The research provides insights into transfusion safety in areas with limited resources which have a high incidence of malaria despite lacking genomic and molecular data. The research delivers critical data which enable local blood transfusion operations and help identify areas for improvement in donor screening procedures and public health programs.

## 6. Limitations

The research findings from this study apply only to the particular hospital where the research was carried out. The study’s statistical power and ability to reach strong conclusions are both restricted by the combination of a small sample size and detection of only five infections. The research findings should be understood as exploratory content which creates new research directions instead of proving existing hypotheses.

## 7. Conclusion

Blood donors showed a low rate of TTIs which occurred during their blood donations. The research requires more extensive multicenter studies to identify risk factors which will improve blood safety measures and develop national transfusion safety guidelines.

## Author Contributions

All authors reviewed this article. Conceptualization: Collince Odiwuor Ogolla, Dr. Benard Guyah, Prof. Apollo Maima, and Benson Amayo. Methodology: Collince Odiwuor Ogolla, Dr. Benard Guyah Prof. Apollo Maima, and Benson Amayo. Formal analysis: Collince Odiwuor Ogolla, and Benson Amayo. Investigation: Collince Odiwuor Ogolla. Data curation: Collince Odiwuor Ogolla. Writing–original draft: Collince Odiwuor Ogolla. Writing–review and editing: Dr. Benard Guyah and Prof. Apollo O. Maima. Supervision: Dr. Benard Guyah and Prof. Apollo O. Maima.

## Funding

This study did not receive any specific funding.

## Disclosure

All authors have read and approved the final version of the manuscript.

## Consent

All authors have given their consent for publication of this article.

## Conflicts of Interest

The authors declare no conflicts of interest.

## Data Availability

The authors confirm that the data supporting the findings of this study are available within the article.
